# FLASH free-electron laser single-shot temporal diagnostic: terahertz-field-driven streaking

**DOI:** 10.1107/S160057751701253X

**Published:** 2018-01-01

**Authors:** Rosen Ivanov, Jia Liu, Günter Brenner, Maciej Brachmanski, Stefan Düsterer

**Affiliations:** a Deutsches Elektronen Synchrotron – DESY, Notkestrasse 85, 22607 Hamburg, Germany; b European XFEL, Holzkoppel 4, 22869 Schenefeld, Germany

**Keywords:** free-electron laser, FLASH, SASE, photon pulse duration, photon pulse arrival time, terahertz streaking

## Abstract

The commissioning of a terahertz-field-driven streak camera installed at the free-electron laser (FEL) FLASH at DESY in Hamburg, being able to deliver photon pulse duration as well as arrival time information with ∼10 fs resolution for each single XUV FEL pulse, is reported. Pulse durations between 300 fs and <15 fs have been measured for different FLASH FEL settings. A comparison between the XUV pulse arrival time and the FEL electron bunch arrival time measured at the FLASH linac section exhibits a correlation width of 20 fs r.m.s., thus demonstrating the excellent operation stability of FLASH. In addition, the terahertz-streaking setup was operated simultaneously to an alternative method to determine the FEL pulse duration based on spectral analysis. FLASH pulse duration derived from simple spectral analysis is in good agreement with that from terahertz-streaking measurement.

## Abstract 2

The installation and commissioning of a pulse length diagnostic setup at FLASH based on terahertz streaking is reported.

## 1. Introduction   

Since FLASH lases in self-amplified spontaneous emission (SASE) mode each photon pulse is ‘unique’ and has a different pulse energy, XUV spectrum and pulse duration (Ackermann *et al.*, 2007 ▸). Furthermore, due to tiny fluctuations in the electron acceleration the arrival time of the XUV pulses jitters in the order of several tens of femtoseconds. The focus of the online photon diagnostics at FLASH is to measure all fluctuating properties as completely as possible and ideally on a shot-to-shot basis. Due to the burst mode structure of FLASH with up to 800 pulses spaced by 1 µs (at a repetition rate of 10 Hz) such measurements are in general challenging.

Several methods have been developed at FLASH and are in use to determine the pulse energy (Tiedtke *et al.*, 2008 ▸), the spectrum (Brenner *et al.*, 2011 ▸, 2016 ▸) and the arrival time of the electron bunches (Angelovski *et al.*, 2012 ▸). However, the XUV pulse duration still lacks an appropriate reliable detector. Several different methods have been pursued (Düsterer *et al.*, 2011 ▸) to identify the optimum pulse duration characterization method to cover the temporal range of 10 fs to a few hundred fs (FWHM) within variable wavelength ranging from 4 to ∼40 nm, and provide sufficient information on all fluctuating temporal properties. For pump–probe experiments which make use of the ultrashort FEL pulses it is necessary to know the pulse duration for a precise data analysis. Precisely characterized XUV pulse duration is also quite important in all kinds of nonlinear interaction studies where XUV intensity plays a vital role. Moreover, when these experiments advance towards improved temporal resolution, they require more and more accurate measurements of the FEL pulse duration. The diagnostics should be non-invasive, thus allowing the experimentalists to maximize the use of the FEL beam for their experimental studies rather than for diagnostics.

The terahertz (THz) streak camera (Grguraš *et al.*, 2012 ▸; Frühling *et al.*, 2009 ▸) has the potential to deliver single-shot pulse duration information essentially wavelength independent and with a high dynamic range (in pulse duration and pulse energy). Furthermore, it is able to be operated with repetition rates up to several hundred kHz (potentially even MHz). It is also capable of providing the arrival time information between the XUV pulse and the THz driving laser for each single pulse with an accuracy well below 10 fs on a shot-to-shot basis. Because of the broad working range (in pulse length, pulse energy, photon energy and repetition rates) the concept can not only be used for FLASH but also for other X-ray FELs (Juranić *et al.*, 2014 ▸; Gorgisyan *et al.*, 2017 ▸), like, for example, the MHz repetition-rate European XFEL and LCLS-II.

## 2. THz streaking setup   

**Figure 1:**
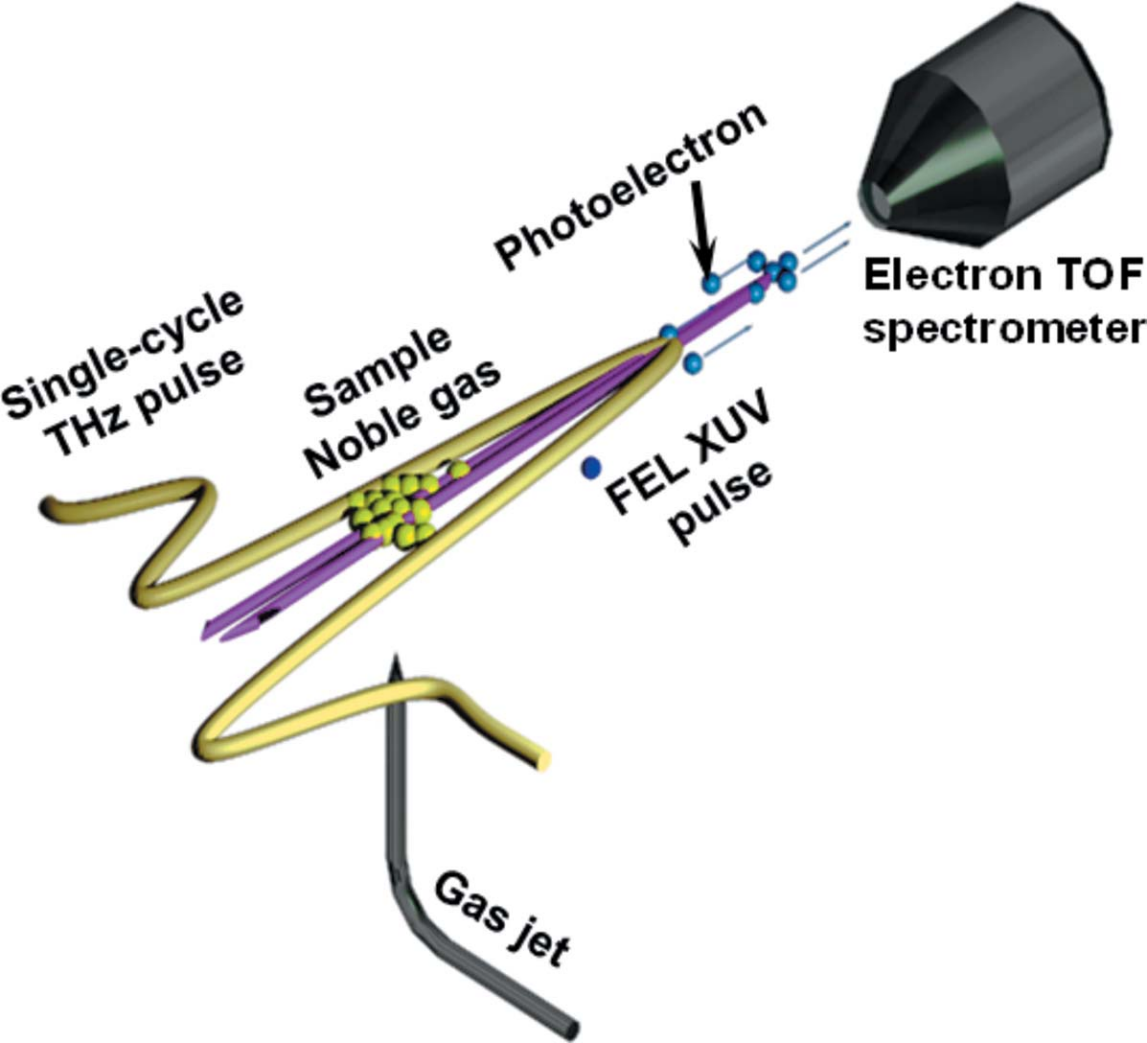
Basic principle of THz streaking. The FEL pulse ionizes noble gas atoms and the resulting photoelectron kinetic energy distribution is detected by an electron time-of-flight (eTOF) detector. The ionization takes place in the presence of a strong linearly polarized THz field influencing the kinetic energy distribution of the photoelectrons. Thus, the XUV pulse profile and arrival time are mapped on the kinetic energy distribution by the THz field.

The THz streak camera uses a noble gas target that is ionized by the FEL pulse (see Fig. 1 ▸). The electrons are then subject to the time-varying electric field of the co-propagating THz field. After interaction with the THz field, the photoelectrons have changed their momentum component in the direction of the field. If the electron wave packet is short compared with the half-period length of the THz field, the temporal structure of the wave packet will be mapped onto the kinetic energy distribution of the emitted electrons and depends on the instantaneous THz vector potential 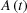 at the precise moment of ionization (Itatani *et al.*, 2002 ▸; Hentschel *et al.*, 2001 ▸). The shift in the kinetic energy relative to the field-free case is 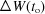 ≃ 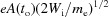, with *W*_i_ the initial kinetic energy of the photoelectron, and *e* and *m*_e_ the electron charge and mass. The measured streaked electron energy spectrum reflects the combined temporal and spectral structure of the FEL pulse. The information about the pulse duration, 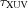 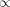 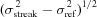, can be extracted from the broadening of the peak measured in the photoelectron spectrum due to the presence of the THz field. Here, 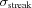 relates to the width of the (streaked) photoelectron line in the presence of the THz radiation while 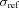 is that of the ‘reference’ (without THz field). The line width also contains information on the FEL bandwidth and the electron time-of-flight (eTOF) detector response. Furthermore, the shift of the streaked photoelectron line provides the arrival time difference between THz pulses and XUV pulses.

The THz streaking setup was built and installed at FLASH1 at the PG0 branch of the high-resolution plane-grating (PG) monochromator beamline (Martins *et al.*, 2006 ▸). FLASH1 is the original FEL line of FLASH, and FLASH2 is a new second FEL line (Faatz *et al.*, 2016 ▸). The PG beamline has the capability to use the non-dispersed zero-order beam as well as higher diffraction orders simultaneously. While the zeroth order of the diffraction grating can be guided to the streaking setup at PG0, the dispersed radiation can be simultaneously used in the PG2 beamline branch to, for example, measure the XUV spectrum with high resolution (Gerasimova *et al.*, 2011 ▸). By measuring the spectral distribution for each FEL pulse we can disentangle the spectral and the temporal contributions. In addition, the FEL pulse duration can in principle be determined by a second-order correlation analysis of the spectrum as shown by Lutman *et al.* (2012 ▸) and Engel *et al.* (2016 ▸).

**Figure 2:**
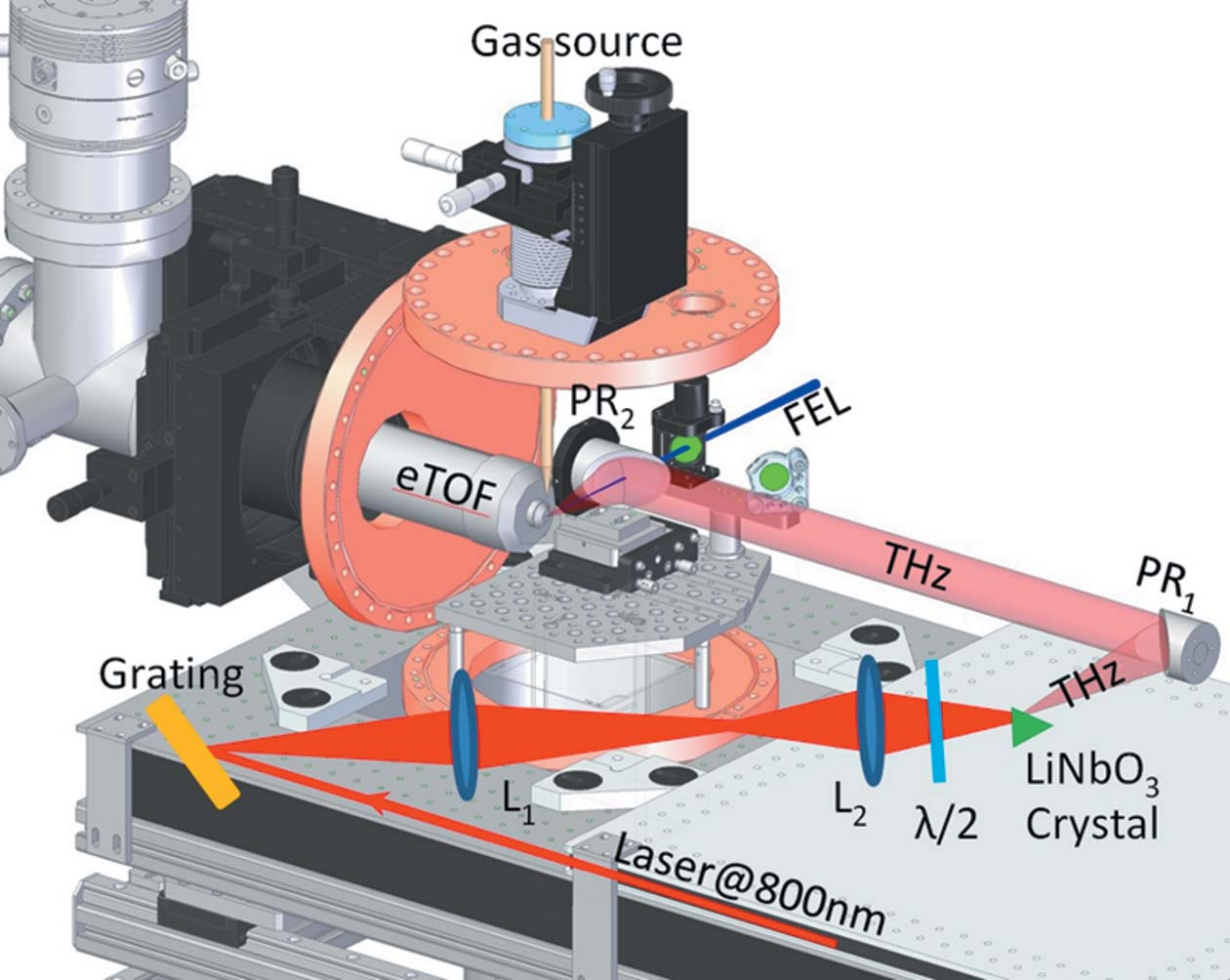
Drawing of the THz streaking experimental setup with the THz generation and transport scheme. The chamber, Cube 250 CF and some of the flanges are not shown in order to give a clearer view.

The experimental THz streaking setup is depicted in Fig. 2 ▸. It consists of a three-axis (*xyz*) movable support structure, an optical breadboard with optics for the THz generation and a compact vacuum UHV chamber (Cube 250 CF). The THz streaking chamber is equipped with a linear eTOF spectrometer mounted on a three-axis manipulator to allow the optimization of the eTOF position with respect to the interaction point. A 90° off-axis parabolic mirror (PR2) with focal length of 101.6 mm is used to collinearly couple in the THz beam and to focus it to the interaction point. XUV is focused by a *f* = 500 mm toroidal mirror through a 3 mm central hole in the parabola to the noble gas target delivered from a gas needle and spatially overlapped with the focus of the THz beam. For the FLASH wavelength range, neon proved the ideal target gas due to the high cross section and simple photoelectron line spectrum. The backing pressure in the chamber was around 1 × 10^−8^ mbar while the pressure in the interaction region was in the low 10^−7^ mbar. In this range no space charge effects were observed. Furthermore, the setup houses different diagnostic tools to determine the temporal and spatial overlap between the FEL and THz pulses. The THz focus was measured to be 2.1 mm FWHM (see below, Fig. 4) and is thus significantly larger compared with the XUV beam focused by the toroidal mirror to a spot size of about 300 µm FWHM.

## 3. THz generation, transport and characterization   

**Figure 3:**
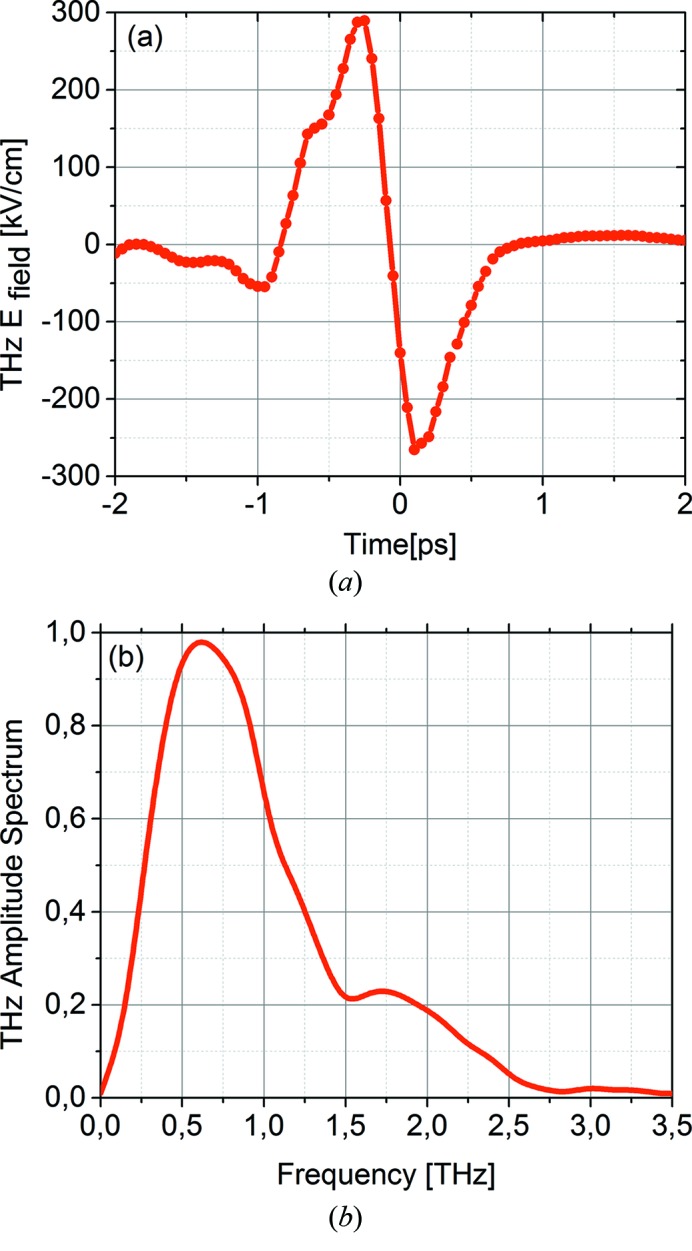
(*a*) THz electric field measured in the interaction region (by THz streaking) and (*b*) the resulting THz spectrum calculated by Fourier transform. The maximum of the spectrum is at 0.6 THz.

The THz radiation was produced by optical rectification of the FLASH1 pump–probe laser pulses (10 Hz, 800 nm, ∼80 fs, 6.5 mJ) using a nonlinear crystal (LiNbO_3_). The process of THz generation can be viewed as the degenerate case of difference frequency generation for identical frequencies 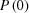 = 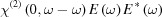. The nonlinear polarization *P* is generated by the incoming field *E* with frequency ω, mediated by the second-order nonlinear susceptibility χ^(2)^. If the laser pulse has a duration of less than 1 ps, the result will be a single-cycle electromagnetic pulse with frequency contents in the terahertz range. To achieve an efficient THz generation process, the phase matching was optimized by a tilted pulse front of the driving pump–probe laser pulse as described by Hebling *et al.* (2002 ▸). We used a setup that consists of a diffractive grating (2000 grooves mm^−1^) and 4*f* two lenses telescope L_1_ (*f* = 150 mm) and L_2_ (*f* = 75 mm) to image the laser pulse onto the nonlinear crystal, providing the required pulse front tilt. The subsequent THz beam transport employed two 90° off-axis gold-coated parabolic mirrors PR_1_ (*f* = 152.4 mm) and PR_2_ (*f* = 101.6 mm). The transport line was kept as short as possible in air to avoid losses and spectral distortions (Fig. 2 ▸). Note that the THz field strength and shape can be directly determined from the streaking spectrogram according to 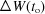 ≃ 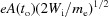. A THz pulse energy of 15 µJ and THz field strength up to 300 kV cm^−1^ were finally achieved in the interaction region. A single-cycle THz pulse with a period of about 3 ps, centered at 0.6 THz, with a vector potential linear slope of at least 500 fs is shown in Fig. 3 ▸. The linear part of the THz field defines the usable time window. The FEL pulse duration including all timing jitters must be in that window, thus the present setup can measure pulse durations of up to 350 fs FWHM. The slope of the THz field, *i.e.* the so-called streaking speed, *s*, is calculated to be 0.1 eV fs^−1^. This leads to a streaking resolution of ∼10 fs (FWHM) for the current THz field (Itatani *et al.*, 2002 ▸; Frühling *et al.*, 2009 ▸).

**Figure 4:**
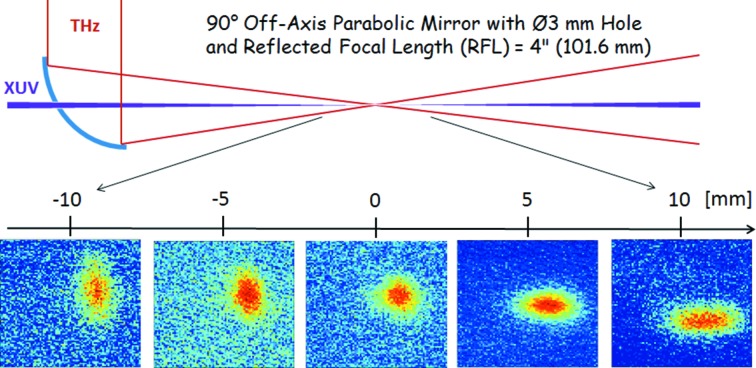
THz beam shape around the interaction point taken with a pyroelectric camera. In the focal plane the THz spot is 2.1 mm FWHM.

The propagation of the THz beam was characterized around the interaction point using a pyroelectric camera (Fig. 4 ▸). A round beam profile of 2.1 mm in FWHM was observed in the focal plane while noticeable astigmatism before and after the focus is observed. Since the phase of the THz field changes within the Rayleigh range, it is important to understand and quantify the additional broadening of the measured photoelectron line due to the fact that electrons are not only collected from an interaction point but rather from a volume (acceptance volume). Within that volume different electrons see different THz field strengths. This leads to a phase shift, the so-called Gouy phase shift defined by 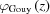 = 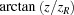. Thus broadening 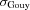 = 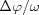 = 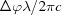 because the Gouy phase has to be taken into account in the data analysis: 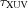 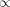 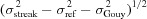 (Frühling, 2011 ▸).

An acceptance ‘volume’ horizontal length of ∼0.5 mm can be estimated by observing the eTOF signal while moving the eTOF spectrometer. As an example, additional broadenings due to the Gouy phase shift are expected to be 13.3 or 10.2 fs (FWHM) when measuring directly at the THz focal plane or 6 mm from the THz focus. The influence of the Gouy phase becomes significant once it is of the same order as the FEL pulse duration. It turned out that the option to move the eTOF with respect to the THz focal spot is very useful in order to adapt the THz setup for different measurement tasks. In addition, the broadening effect can be weakened by minimizing the acceptance volume (*e.g.* by applying a smaller diameter gas needle). For instance, the above values reduce to 6.3 fs and 4.8 fs (FWHM) if the acceptance volume is 0.25 mm. Another possibility to minimize the Gouy phase induced broadening is to increase the THz beam size by using a longer focal length parabola at the expense of reducing the THz field strength which will negatively affect the resolution.

## 4. Experimental results   

**Figure 5:**
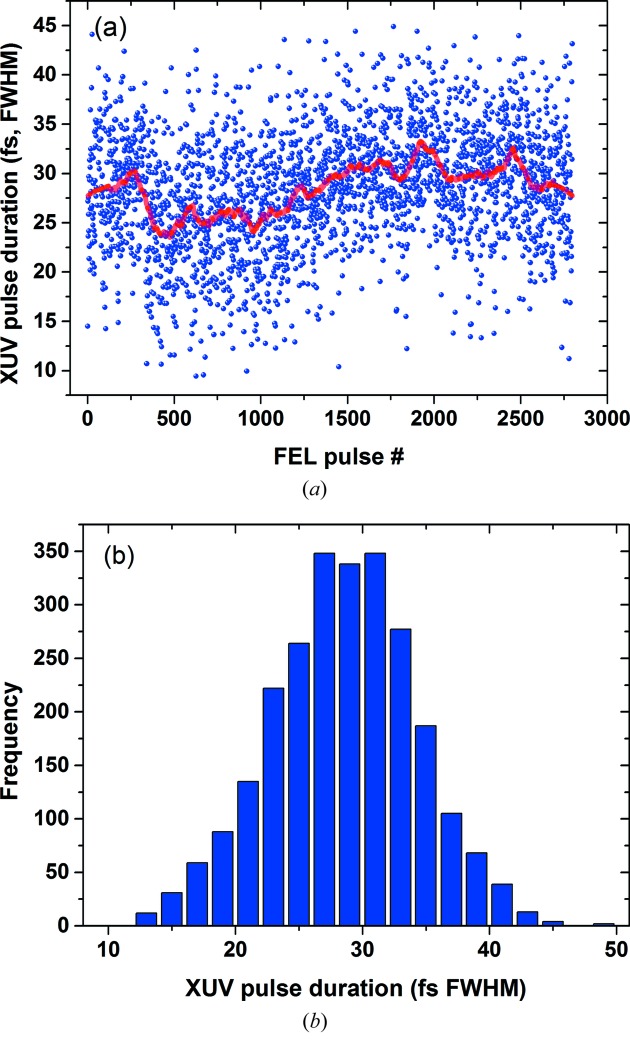
(*a*) Single-shot pulse duration for ∼3000 FLASH shots. The red line indicates the mean value (∼28 fs) [error bars (not shown) due to the fit uncertainty are of the order of 20%], and (*b*) a histogram of the measured pulse durations.

Up to now several different FEL operation settings have been used to commission the technique over a wide range of pulse durations from 350 fs to less than 15 fs (FWHM). This was achieved by varying the electron bunch charge from 0.44 to less than 0.1 nC for FEL wavelengths of 7, 13 and 20 nm. For each setting the FLASH single-shot pulse duration as well as the arrival time of the XUV pulses with respect to the THz generating optical (pump–probe) laser were measured. The FLASH1 pump–probe laser is optically synchronized to the FLASH master clock (Schulz *et al.*, 2015 ▸). As one example, a recorded time sequence of 5 min (3000 pulses) is shown in Fig. 5 ▸; clear inherent fluctuation of the pulse duration around the measured mean value of ∼28 fs (FWHM) is observed.

**Figure 6:**
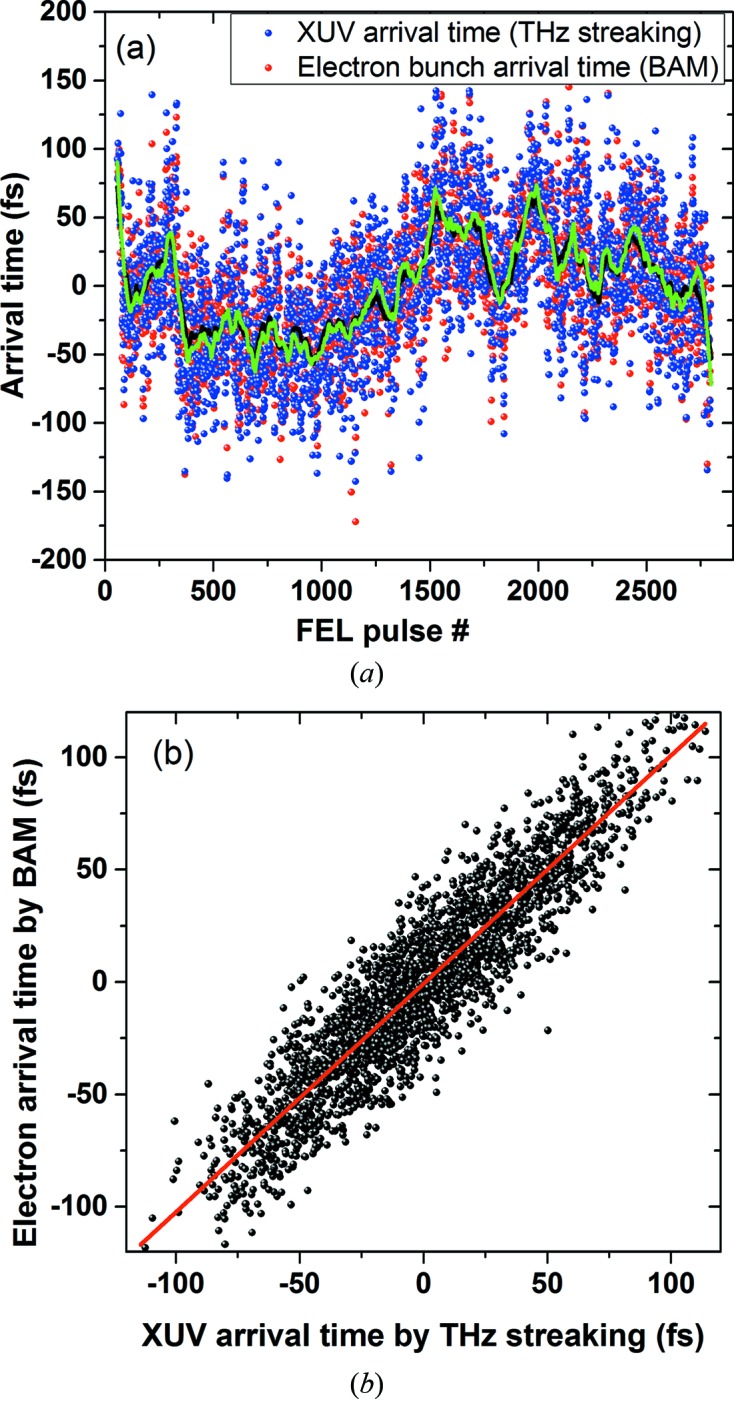
(*a*) Arrival time plotted for the same FEL shots as in Fig. 5 ▸. The XUV (blue) and electron (red) arrival times agree well on a shot-to-shot basis (dots). Averaging the arrival time over 10 s (lines, green for THz streaking data and black for BAM data) still provides a very good agreement. (*b*) Correlation plot comparing the arrival time measured for electrons (BAM) and the XUV photon pulses at the experiment (streaking) showing a correlation width of only 20 fs r.m.s. The red line indicates the linear fit.

As a second important result of the commissioning runs we could verify the assumption that the high precision of the electron beam arrival time monitor (BAM) measuring the arrival time of the electron bunches holds also for the arrival time of the XUV pulses in all measured cases. So far this relation has only been demonstrated once for a specific case (Schulz *et al.*, 2015 ▸). We determined a very good agreement of the arrival time (with respect to the FEL master timing) of the electrons – measured at around 200 m upstream of the experimental hall in the accelerator tunnel – and the arrival time of the XUV pulses with respect to the pump–probe laser (producing our THz) at the experiment. The shot-to-shot correlation shown in Fig. 6 ▸ reveals a correlation width of less than 20 fs r.m.s. for most of the settings investigated so far. This result tells us that one can indeed significantly improve the time resolution of optical–XUV pump–probe experiments by sorting the acquired data according to the electron bunch arrival times measured by the BAM (Savelyev *et al.*, 2017 ▸). Moreover, this diagnostic can in future be used to characterize and improve the performance of the FLASH FEL synchronization system and the arrival time detector.

**Figure 7:**
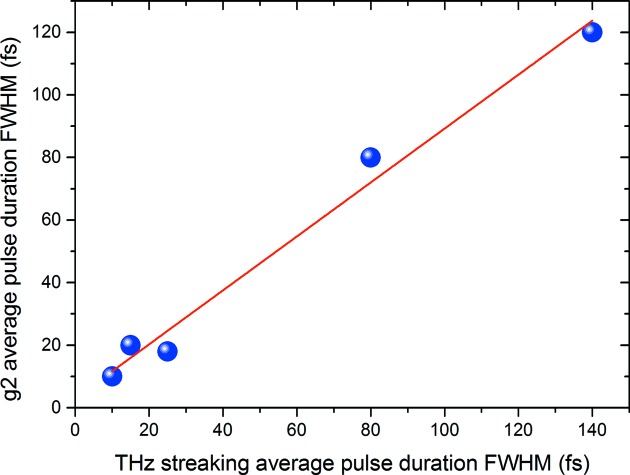
Pulse durations determined by the analysis of the second order [*g* ^(2)^] spectral correlation and pulse durations taken from the THz streaking agree quite well and demonstrate the applicability of the *g* ^(2)^ method over a large range of pulse durations.

In addition to the single-shot SASE FEL pulse durations and arrival times measured by the THz streaking setup at PG0, high-resolution XUV spectra can be recorded simultaneously for the exact same FEL pulse. This allows several indirect methods attempting to infer the FEL pulse duration from the spectral distribution to be applied. One rather simple approach is based on counting the spectral spikes which are to a certain extent proportional to the temporal SASE pulse duration (Krinsky & Gluckstern, 2003 ▸). More advanced methods with a presumably larger applicable range are based on one- and two-dimensional second-order spectral correlation [*g* ^(2)^] analysis (Engel *et al.*, 2016 ▸; Gorobtsov *et al.*, 2017 ▸). Fig. 7 ▸ shows a first result of the comparison between the pulse duration determined by THz streaking and the one-dimensional *g* ^(2)^ method for different FEL settings. The analysis for the *g* ^(2)^ approach is explained in detail by Lutman *et al.* (2012 ▸). The measurements show a good agreement and are promising in that the applicability range of the spectral analysis is indeed rather large and can be used as a simple way to estimate the XUV pulse duration. A publication describing the different approaches and experimental findings in more detail is in preparation

## 5. Conclusion   

We report on the installation and commissioning of a pulse-length diagnostic setup at FLASH1 based on THz streaking. We have demonstrated single-shot pulse duration measurements of XUV pulses covering the full range of <15 fs to 350 fs. In addition, we verified the excellent agreement of the electron beam arrival time monitor (BAM) data with the actual arrival time fluctuations of the pump–probe laser at the FLASH1 experimental endstations. A comparison of methods which determine the XUV pulse durations indirectly by spectral analysis shows good agreement with the pulse durations determined by THz streaking measuring in parallel. The THz streaking technique allows us to measure the single-shot SASE FEL pulse duration in the presence of significant arrival time fluctuations (sometimes even bigger than the FEL pulse duration). One can easily imagine how the analysis of user experiments can be improved if such pulse-resolved online photon diagnostic data are available.

The setup is currently redesigned to fit at FLASH2 into a beamline section in front of all experimental endstations to provide pulse-resolved online photon pulse duration measurements in the future.
